# Comparison of Pain Scores in Postoperative Patients: Intravenous Morphine Patient-Controlled Analgesia vs Iontophoretic Transdermal Fentanyl

**DOI:** 10.7759/cureus.752

**Published:** 2016-08-26

**Authors:** Gabriel D Glaun, Anthony M Caram, Nirav Patel, Hayden M Sandler

**Affiliations:** 1 University of Central Florida College of Medicine, University of Central Florida College of Medicine, Orlando, FL

**Keywords:** postoperative pain control, fentanyl, iontophoretic transdermal system, morphine, surgery, pain-controlled analgesia, orthopedic surgery

## Abstract

Postoperative management of pain has traditionally utilized intravenous (IV) morphine for pain control. An alternative approach to the invasive patient-controlled analgesia (PCA) system is the administration of transdermal analgesics, such as fentanyl. In 2006 the Food and Drug Administration (FDA) approved the fentanyl hydrochloride (fentanyl HCl) iontophoretic transdermal system (ITS), which utilizes iontophoretic technology to produce a controlled electrical current that propels ionized fentanyl molecules into the systemic vasculature. Transdermal fentanyl has been shown to be equivalent or superior to IV morphine PCA in a variety of postoperative settings with patients experiencing decreased pain scores and a favorable side effect profile.

## Introduction and background

Postoperative management has traditionally utilized intravenous (IV) morphine for pain control. A commonly utilized method for administration of IV morphine is via patient-controlled analgesia (PCA). PCA provides patients with autonomy, as patients can self-administer small loading doses at predetermined rates, which also allows synergy between adequate pain control and avoidance of side effects observed in high-dose boluses of opioids [1]. However, the literature has cited cases of PCA pump failure, programming errors, and tinkering of the device by patients, which can jeopardize patient well-being and lead to overdose-related respiratory depression and death [1]. Further, patients have expressed disinterest in wearing the bulky PCA pump, which limits mobility, especially during the fragile postoperative period [1].

An alternative approach to the invasive PCA system is the administration of transdermal analgesics. This pain control method has gained popularity among patients, as it avoids the risk of needle-induced injuries and their sequelae [2-3]. The absorption of transdermal analgesics occurs via passive diffusion. Thus, the medicine is administered slowly and in a continuous fashion that is ideal for chronic pain relief. However, in the postoperative, acute pain setting, this mode of analgesic relief is insufficient [4].

In 2006, the Food and Drug Administration (FDA) approved the fentanyl HCl iontophoretic transdermal system (ITS). This novel pain control modality utilizes iontophoretic technology to produce a controlled electrical current that propels ionized fentanyl molecules into the vasculature, as opposed to just relying on passive diffusion [4]. Utilizing iontophoresis, patients have the capacity to receive on-demand doses, unlike previously used transdermal analgesics. Fentanyl ITS delivers a fixed amount of opioids, virtually eliminating the risk of manual programming risks reported in PCAs [4]. The electro-propulsion unique to the fentanyl ITS system allows this modality to be utilized for acute postoperative pain management.

As is the case with morphine PCA, fentanyl ITS does carry a risk of adverse effects. The iontophoresis may induce a transient abnormal sensation at the application site of the transdermal patch [5]. The technology also carries the risk of causing skin erythema beneath the electrodes, which typically resolves over a 24-hour period [6].

While progress has been made in the past few decades regarding postoperative pain management, the literature reports insufficient management on a global scale [7]. Analgesics and their mode of application selected for postoperative pain management are provider-specific. This article will aim to compare and contrast the adverse effects and efficacy of IV morphine PCA and fentanyl ITS with regard to pain control in the postoperative period following various surgical procedures.

## Review

### Methods

An electronic database search of titles and abstracts was conducted on PubMed and Google Scholar. The keywords that were used in the search included “fentanyl,” “iontophoretic transdermal system,” “morphine,” “surgery,” “postoperative,” and “pain.” Articles that were relevant to postoperative pain scores and side effects of fentanyl and morphine were included in the initial review. These articles were then further narrowed down to include only those studies that compared iontophoretic transdermal fentanyl to intravenous morphine. All studies evaluated in this review were randomized control trials.

### Review

Postoperative pain is a common complaint among surgical patients. IV morphine PCA is a common regimen given to patients, but it is not without side effects [8]. Fentanyl ITS is becoming an effective option for postoperative pain. Recently, studies have shown clinical efficacy and even superiority in using fentanyl ITS compared to IV morphine PCA [1-3, 9-10].

Saffer, et al. performed a meta-analysis of two randomized controlled trials comparing fentanyl ITS versus IV morphine PCA in postoperative management of gynecological surgery [9]. The baseline demographics were similar for fentanyl ITS and IV morphine PCA groups. A total of 604 patients were included in the study, with a mean age of 45 years, and a mean BMI of 29. The majority of patients were Caucasian (65%). The patients were asked to complete a postoperative pain assessment on a four-point scale of “excellent,” “good,” “fair,” or “poor.” A final assessment was completed by investigators, who used the same four-point scale to assess the two methods of pain control. Ultimately, the data showed that significantly more patients rated the pain control as “excellent” in the fentanyl ITS group at the 24-hour postoperative assessment (49.3 versus 37.4% (*p* = 0.0029), respectively) as did the investigators in their final assessments (59.5 versus 38.0% ( *p* < 0.0001), respectively) compared to the IV morphine PCA group [9].

A pooled analysis of two randomized controlled trials by Hartrick, et al. showed similar statistically significant superiority in postoperative pain scores in patients undergoing orthopedic surgeries [10]. A total of 590 patients received fentanyl ITS and 626 patients received IV morphine PCA for up to 72 hours. The mean age was approximately 60 years and the patients were mostly Caucasian (90.5%). More than 80% of the patients underwent hip replacement. The efficacy measures were included in the patient global assessment and the investigator global assessment, which utilized a four-point scale of “excellent,” “good,” “fair,” or “poor.” More patients rated the pain control as “excellent” in the fentanyl ITS group at the 24-hour postoperative assessment (44.8 versus 33.0% (*p* < 0.001), respectively), at the 48-hour postoperative assessment (37.5 versus 25.3% (*p* < 0.001), respectively), as well as at the 72-hour postoperative assessment (54.3 versus 39.6% (*p* < 0.001), respectively), compared to the IV morphine PCA group [10]. More investigators during their final assessment rated postoperative pain control as “excellent” in the fentanyl ITS group (57.4 versus 36.9% (*p* < 0.001), respectively) compared to the IV morphine PCA group [10].

While studies did reveal positive results of using iontophoretic transdermal fentanyl, not all studies showed such clear cut superiority. Rather, they demonstrated that transdermal fentanyl was not significantly better in improving postoperative pain compared to IV morphine PCA, but had comparable effects on pain scores and adverse effects in a variety of different surgical fields [1-3]. Also, these studies were prospective randomized controlled studies, whereas the articles evidencing fentanyl’s superiority were meta- or pooled analyses.

A prospective, randomized, controlled parallel group study by Viscusi, et al. demonstrated comparable effects of fentanyl ITS and morphine PCA [1]. A total of 234 participants completed the study for the fentanyl ITS group and 240 completed the study for the PCA with morphine group [1]. The participants in this study were most commonly Caucasian (73.7 and 73.1% for the fentanyl ITS and morphine PCA groups, respectively) and mostly females (72.5 and 74.4% for the fentanyl ITS and morphine PCA groups, respectively). The majority of the surgeries were lower abdominal (55.7 and 57.8% for the fentanyl ITS and morphine PCA groups, respectively) or orthopedic surgeries (36.7 and 34.7% for the fentanyl ITS and morphine PCA groups, respectively) [1]. The dosing was qualitatively similar between the fentanyl ITS and PCA with morphine systems [1]. Morphine PCA pumps delivered 1 mg boluses with five-minute lockouts, up to 10 doses per hour, and the fentanyl ITS provided 40 micrograms over 10 minutes, up to 80 doses per 24 hours [1]. The primary end point of this study was a patient global assessment taken at 24 hours, and then 48 and 72 hours if the patients remained in the study [1]. The patients were asked to rate their pain scores within the categories of “excellent,” “good,” “fair,” or “poor” [1]. The distribution of pain scores was not statistically significantly different between the study groups [1]. The combined pain score ratings at 24 hours of “good” or “excellent” were also not statistically significantly different, which for the fentanyl ITS was 73.7% of patients and 76.9% for the PCA with morphine group (*p* = .36) [1]. The fentanyl ITS and morphine PCA groups in this study had similar adverse effects of nausea, headache, vomiting, and prutitis [1].

Minkowitz, et al. performed a randomized, active-controlled, parallel group, multicenter study of 506 patients and also demonstrated comparable effects between fentanyl ITS and morphine PCA [2]. The patients were mostly female (84.1 and 83.9% for the fentanyl ITS and morphine PCA groups, respectively). They underwent pelvic (63.1 and 68.1% for the fentanyl ITS and morphine PCA groups, respectively) or abdominal surgeries (36.9 and 31.9% for the fentanyl ITS and morphine PCA groups, respectively) [2]. A total of 252 patients received fentanyl ITS and 254 patients received PCA with morphine [2]. The qualitative dosing of the fentanyl ITS and morphine PCA systems was similar [2]. Morphine PCA pumps delivered 1 mg boluses, a five-minute lockout between doses, and a maximum of 10 doses per 24 hours [2]. The fentanyl ITS system provided 40 micrograms over 10 minutes up to six doses per hour, or a maximum of 80 doses per 24 hours [2]. The primary outcome measure was a patient global assessment that asked patients to rate their pain scores within the categories of “excellent,” “good,” “fair,” or “poor” [2]. At 24 hours, 84.9% of patients in the fentanyl ITS group and 84.3% of patients in the morphine PCA group rated their pain scores as “good” or “excellent” (*p* = .835) [2]. The pain score distribution was similar among both groups [2]. This study also showed that pain scores were not statistically significant in relation to the BMI of patients [2].

Grond, et al. performed a multicenter, prospective, open-labeled, randomized, parallel, controlled study that also exhibited comparable efficacy between fentanyl ITS and morphine PCA in postoperative patients [3]. A total of 325 patients were randomized into the fentanyl ITS group and 335 patients into the morphine PCA group [3]. The patient baseline characteristics were not statistically significantly different [3]. The patients were mostly female (57.2 and 57.0% for the fentanyl ITS and morphine PCA groups, respectively), with mean ages of 52.5 and 53 years for the fentanyl ITS and morphine PCA groups, respectively. The patients underwent a variety of surgical procedures. For the fentanyl ITS group, 31.7% underwent lower abdominal surgery, 24.3% underwent orthopedic-lower extremity surgery, 12.9% underwent pelvic surgery, 13.8% underwent upper abdominal surgery, and 17.2% underwent other surgeries [3]. For the morphine PCA group, 27.5% underwent lower abdominal surgery, 33.1% underwent orthopedic-lower extremity surgery, 11.6% underwent pelvic surgery, 10.4% underwent upper abdominal surgery, and 17.3% underwent other surgeries [3]. The fentanyl ITS was programmed to deliver 40 micrograms over 10 minutes, or up to 80 doses per 24 hours [3]. The morphine PCA pumps were programmed differently across the hospitals but allowed up to 20 mg per two hours, or a maximum of 240 mg per 24 hours [3]. The primary outcome measure was a patient global assessment in which the patients were asked to rate their pain scores within the categories of “excellent,” “good,” “fair,” or “poor” at 24 hours after the initiation of therapy [3]. The pain scores were rated as “good” or “excellent” by 86.2% of patients in the fentanyl ITS group, whereas pain scores were rated as “good” or “excellent” by 87.5% of patients in the morphine PCA group (CI, -6.5 to 3.9%) [3]. There were also no statistically significant differences at 48 or 72 hours after initiation of treatment [3].

Regardless of the mixed evidence in the literature, morphine has well-documented adverse effects. Morphine has shown to cause respiratory depression, pruritus, urinary retention, and nausea more frequently when compared to other narcotics [8, 11]. Further, the fentanyl ITS uses a needle-free approach, avoiding injuries to staff and discomfort to patients [2-3].

## Conclusions

Fentanyl ITS has been shown to be as effective or even more effective than IV morphine PCA in a variety of postoperative settings. This reason plus fentanyl’s seemingly favorable side effect profile and lack of needle administration leads us to believe that further investigation may be warranted to establish fentanyl ITS as a viable alternative to the traditional IV morphine PCA in the management of postoperative care in a variety of surgical settings.
